# Abdominal Cocoon Secondary to Meconium Peritonitis in a Neonate: A Case Report

**Published:** 2013-01-01

**Authors:** Safwan Ahmad, Kanchan Kayastha, Sana Javed, Arsalan Wasti

**Affiliations:** Department of Pediatric Surgery, The Children’s Hospital and the Institute of Child Health Lahore, Pakistan

**Keywords:** Abdominal cocoon, Neonatal intestinal obstruction, Meconium peritonitis

## Abstract

Abdominal cocoon is a complete or partial encasement of intestines and rarely viscera by a fibrocollagenous sac which is usually formed by a nonspecific chronic inflammatory reaction. We report a case of abdominal cocoon in a 2-day-old neonate presenting with intestinal obstruction.

## INTRODUCTION

Abdominal cocoon is a complete or partial encasement of intestines and rarely viscera by a fibrocollagenous cocoon like sac which is usually formed by a nonspecific chronic inflammatory reaction [1-3].The condition has frequently been described in females of tropical and subtropical regions around their puberty [2]. The etiology is pointed towards chronic peritonitis due to tuberculosis or ascending infection in females [3, 4]. Meconium peritonitis has never been reported as an etiology of abdominal cocoon. Our case adds another cause to the list of etiologies and favors secondary causes instead of congenital causes which lead to formation of abdominal cocoon.

## CASE REPORT

A 2-day-old male neonate, born by spontaneous vaginal delivery at home, presented with failure to pass meconium, vomiting and abdominal distention since birth. The history of polyhydramnios was present. Antenatal anomaly scan was reported normal. On general physical examination, patient had weak cry, feeble peripheral pulses, and respiratory rate of 42 per minute. The birth weight was only 2.5 kg. On examination, abdomen was symmetrically distended with tenderness. Routine lab tests were normal. The abdominal x-ray (Fig. 1) showed bowel gas shadows gathered in the center of abdomen and seemed to be surrounded by homogenous opacity. The abdominal ultrasound showed free fluid present in peritoneal cavity. We suspected complicated meconium ileus preoperatively.

**Figure:**
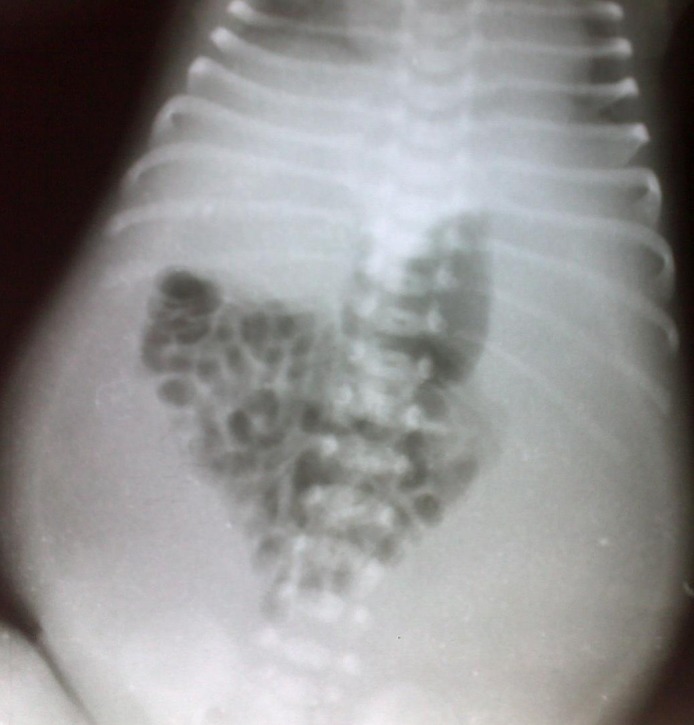
Figure 1: Abdominal radiograph.

On exploration, there was about 300 ml of meconium stained fluid in the peritoneal cavity. Entire small bowel, large bowel, and liver were encased in a transparent membrane (Fig. 2). 

**Figure:**
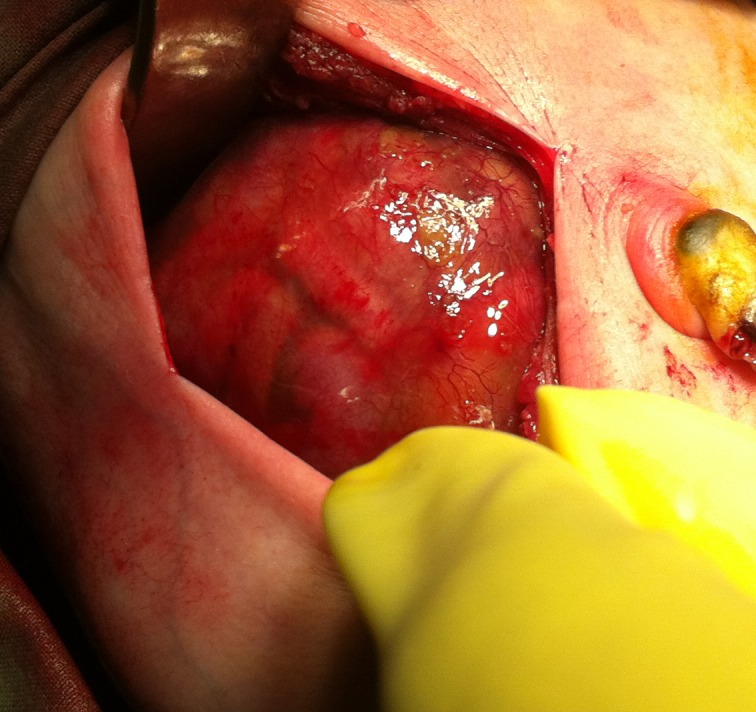
Figure 2: Entire bowel encased in the cocoon.

**Figure:**
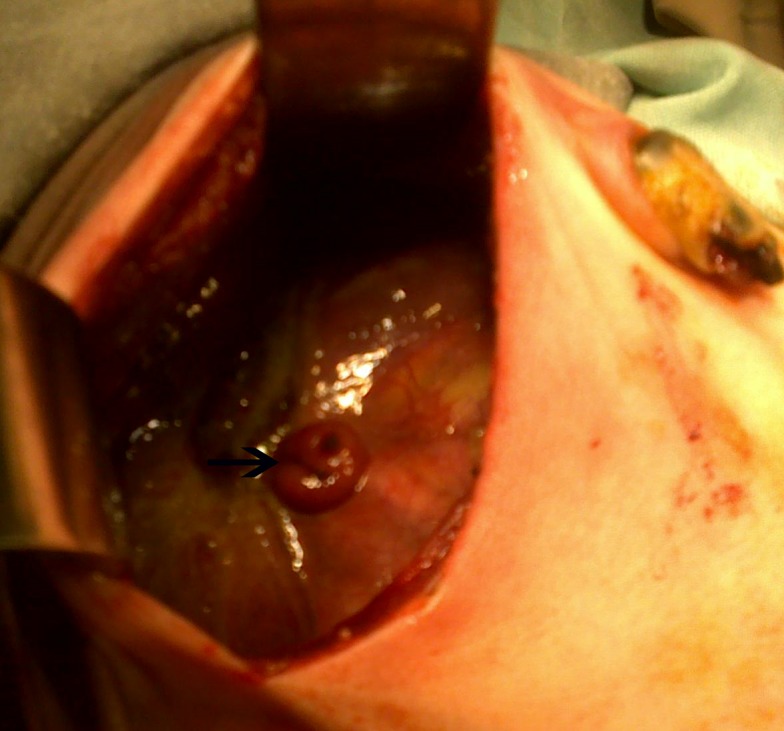
Figure 3: Perforated ileum pouting like a stoma.

**Figure:**
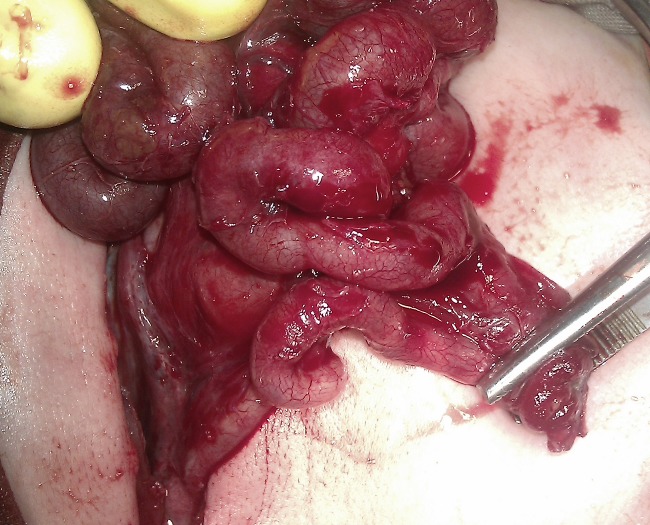
Figure 4: Mobilized bowel and perforated ends in forceps.

The distal ileum had a perforation which was pouting like a stoma through the cocoon (Fig. 3). The membrane was dissected off the intestines. Moreover, inter-loop adhesionolysis was also performed (Fig. 4). Distal patency of bowel was confirmed and ileoileal anastomosis performed after refreshing the margins of the perforation. 

The immediate postoperative recovery was uneventful. The nasogastric tube was removed on 4th postoperative day. Patient was allowed orally on the same day and discharged on establishing full feeds on 8th POD. The patient again presented on 10th postoperative day with anastomotic leakage. He underwent re-exploration and divided ileostomy was formed. However, the neonate succumbed to sepsis on 13th postoperative. The histopathology of the membrane showed proliferation of fibrocytes with enrichment of collagen fibers having non-specific inflammatory reaction.

## DISCUSSION

Abdominal cocoon may be idiopathic or acquired. Chronic inflammations due to tuberculosis, systemic lupus erythrematosis and sarcoidosis, echo virus peritonitis, retrograde infection in females, peritoneal dialysis (staphylococcal, pseudomonas), following placement of LeVeen shunt and ventriculoperitoneal shunt, drugs like beta blocker practolol, and tumors like leiomyoma, endometrial cyst or ovarian tumors are the proposed acquired etiologies of abdominal cocoon [1-7,9]. 

Congenital etiology has also been emphasized and it is considered that cocoon like membrane may be derived from yolk sac during 12th week of gestation or omentum plays a role in the formation of abdominal cocoon [5]. We believe that meconium peritonitis led to the formation of abdominal cocoon in our case. There are three varieties of abdominal cocoon. In type I, membrane covers a part of intestine, in type II entire intestine, and in type III other viscera along with entire intestine are encased [7]. Our case was a type III abdominal cocoon.

The clinical presentation is often non contributory to the clinical suspicion, as in our case, where the patient presented with neonatal intestinal obstruction. Older patients may have abdominal pain, with or without constipation or abdominal mass. 

Intestinal obstruction with peritonitis may develop secondary to myriad of etiologies and a high index of suspicion is required to detect this condition as to extreme scarcity of abdominal cocoon especially in neonates and infants. The disease has been reported to remain dormant till the old age and found at operation done for other reasons [6]. 

The definitive treatment is dissection and excision of the membrane [1-6]. Some authors also recommend appendectomy as part of operation fearing a difficult dissection on account of adhesions should the patient has appendicitis in future [6]. 

To conclude, abdominal cocoon is extremely rare in neonates and infants. Crowding of bowel gas shadows with peripheral homogenous opacity on plain radiograph may raise a suspicion of abdominal cocoon. Meconium peritonitis may be enlisted in the etiology of abdominal cocoon.

## Footnotes

**Source of Support:** Nil

**Conflict of Interest:** None
